# Zingerone as a neuroprotective agent in experimental diabetes: evidence from oxidative stress, inflammatory, and apoptotic markers

**DOI:** 10.1007/s00210-026-05268-y

**Published:** 2026-04-16

**Authors:** Ferit Kansu Ornek, Gungor Cagdas Dincel, Feyza Basak, Emin Sengul, Metin Kiliclioglu, Serkan Yildirim

**Affiliations:** 1 Department of Pathology, Faculty of Veterinary Medicine, Ataturk University, Erzurum, Turkey; 2 Department of Medical Pathology, Faculty of Medicine, Ankara Medipol University, Ankara, Turkey; 3 Department of Histology and Embryology, Faculty of Medicine, Karabuk University, Karabük, Turkey; 4 Department of Physiology, Ataturk University, Erzurum, Turkey; 5 Department of Pathology, Faculty of Veterinary Medicine, Kyrgyzstan-Turkey Manas University, Bishkek, Kyrgyzstan

**Keywords:** Apoptosis, Diabetes mellitus, Neuroprotection, Neurotoxicity, Oxidative damage, Zingerone

## Abstract

Diabetes mellitus (DM) is a global disease that directly affects many organs, including the brain and cerebellum. This study aims to evaluate the effects of zingerone (ZO) on neurotoxicity, a prevalent consequence of DM. Female Sprague–Dawley rats (*n* = 60) were divided into 6 groups. Experimental groups were established as Control, DM, DM + Metformin (MET), DM + ZO25, DM + ZO50, and ZO50. At the end of 28 days, the brain and cerebellum tissues were used for the analyses. To evaluate oxidative stress and oxidative DNA damage, neuronal nitric oxide synthase (nNOS), inducible nitric oxide synthase (iNOS), 8-hydroxy-2′-deoxyguanosine (8-OHdG), malondialdehyde (MDA), glutathione (GSH), superoxide dismutase (SOD), and glutathione reductase (GR) levels were measured. Apoptosis was assessed by determining Caspase-3 expression levels. In addition, H2A.X, NeuN, and glial fibrillary acidic protein (GFAP) expression levels were analyzed to evaluate DNA damage, neuronal integrity, and glial activation, respectively.While nNOS, 8-OHdG, Caspase-3, H2A.X, NeuN, GFAP, MDA, and iNOS levels increased in the DM group, it was observed that tissue damage decreased in a dose-dependent manner in the ZO treatment groups. Also, while there was a decrease in SOD and GR activity and GSH levels in the DM group, ZO 50 mg/kg treatment caused an increase in these enzymes. This study suggests that ZO may exert neuroprotective effects by mitigating oxidative stress, suppressing inflammation, and modulating apoptosis in diabetes-induced neurotoxicity.

**Graphical Abstract:**

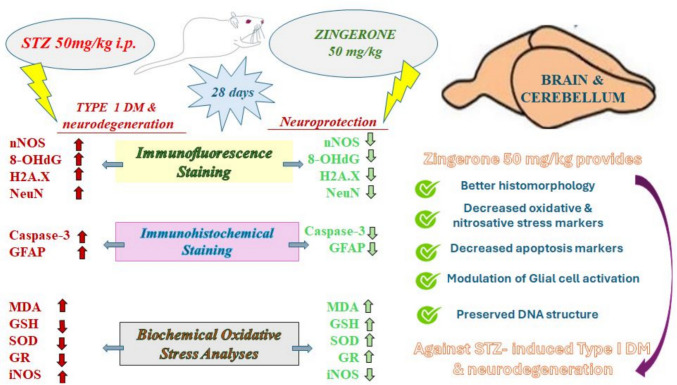

## Introduction

Diabetes mellitus (DM), one of the most common chronic diseases, is an endocrine and metabolic condition that alters glucose, lipid, and protein metabolism (Antar et al. 2023). DM develops due to three factors: pancreatic β-cell dysfunction, insulin resistance in peripheral cells, and an increase in hepatic glucose production, all leading to chronic hyperglycemia (Balaji et al. 2019). Chronic hyperglycemia causes serious health problems such as neuropathy, retinopathy, and nephropathy and especially carbohydrate, fat, and protein metabolism disorders (Islam et al. 2025). Diabetic neurodegeneration is one of the most common chronic consequences of type 1 (T1DM) and type 2 (T2DM) diabetes mellitus, affecting 50% of individuals (Bahadoran and Ghasemi 2025).

Oxidative stress is acknowledged as a significant contributor to the detrimental consequences of diabetes on the central nervous system (Caturano et al. 2023). Chronic hyperglycemia disrupts the mitochondrial respiratory chain, leading to electron leakage and excessive generation of reactive oxygen species (ROS) (Iheagwam et al. 2025). Metabolic processes, such as the development of advanced glycation end products (AGEs), elevate the production of ROS, resulting in heightened intracellular oxidative stress (Zhang et al. 2025). Neural tissue is particularly susceptible to oxidative injury due to its elevated oxygen utilization, prevalence of unsaturated fatty acids, and inadequate antioxidant defenses (Liu et al. 2017). Thus, heightened concentrations of lipid peroxidation products, such as MDA, augmented DNA oxidation indicators, including 8-OHdG, and diminished levels of antioxidant defense enzymes, such as SOD, GSH, and GR, are regarded as significant biochemical biomarkers of neurotoxicity in diabetes (Yang et al. 2011).

On the other hand, nitrosative stress is a critical contributor to cerebral damage in diabetes (Mazumdar and Singh 2024). Nitric oxide (NO), generated by different isoforms of nitric oxide synthase (NOS), functions as a crucial signaling molecule but can convert into a harmful toxin when excessively produced (Akanji et al. 2020). The overactivation of nNOS impairs mitochondrial function in neurons, leading to the generation of peroxynitrite, whereas the excessive release of iNOS by inflammatory cells exacerbates chronic inflammation. The concurrent activation of these two pathways results in markedly elevated oxidative and nitrosative stress within the diabetes-induced central nervous system (Radi et al. 2002).

Apoptosis is a crucial component of diabetic neurodegeneration (Tolkovsky 2002). Hyperglycemia-induced ROS/RNS undermines mitochondrial membrane integrity and prompts the release of cytochrome c, hence beginning caspase-dependent apoptosis (Russell et al. 2002). Increased levels of Caspase-3 are a significant marker of heightened programmed cell death in the brain tissue of diabetics (Jafari Anarkooli et al. 2008). Increased H2A.X expression, a biomarker for DNA double-strand breaks, signifies considerable compromise of brain genomic integrity (Weyemi et al. 2018). DM further promotes the activation of glial cells (Llorián-Salvador et al. 2024). Reactive astrocytes exhibit elevated glial fibriller acidic protein (GFAP) levels, leading to enhanced neuroinflammatory processes. This inflammatory response can lead to disruption of neuronal integrity and damage, particularly in regions such as the cerebellum and brain (Adamu et al. 2024).

Given these complex pathophysiological mechanisms, it is increasingly recognized that single-target approaches may be insufficient for the treatment of diabetic neurodegeneration. Consequently, researchers have focused on pharmacological compounds derived from natural sources with a diverse spectrum of effects. Phenolic compounds are natural chemicals that have gained importance in recent years due to their antioxidant, anti-inflammatory, and anti-apoptotic properties (Panzella 2020). These compounds have been reported to neutralize free radicals and activate the endogenous defense system, providing benefits in various diseases such as diabetic central nervous system damage (Rahman and Rahaman 2021). Zingerone (ZO), a bioactive phenolic molecule derived from the root of *Zingiber officinale* (ginger), has attracted attention due to its reported antioxidant properties (Rossi et al. 2025). The hydroxyl and methoxy groups in the molecule’s chemical structure enhance free radical scavenging ability and disrupt oxidative chain reactions (Wali et al. 2020). Studies show that ZO reduces lipid peroxidation, enhances antioxidant enzymes such as SOD, CAT, and GSH-Px, attenuates inflammation-related signaling pathways such as iNOS, and inhibits apoptotic mechanisms, preserving mitochondrial membrane integrity and improving cellular energy balance (Wang et al. 2025). While detailed evidence of blood glucose reduction from direct animal models or systematic measurements in clinical studies is lacking, other studies in the literature highlight the beneficial effects of ZO on hyperglycemia in diabetic animal models (Alshathly 2019).

Despite the widespread prevalence and global effects of DM, the effect of ZO on the etiology of diabetic neurodegeneration and neurotoxicity remains to be elucidated. Numerous animal models have investigated the neuroprotective effects of ZO; however, comprehensive studies simultaneously assessing oxidative stress, nitrosative stress, glial activation, and apoptosis in diabetic brain and also cerebellar tissues are limited. Given the critical roles of the cerebellum, investigating the susceptibility of cerebellar tissue to diabetes in conjunction with the brain is increasingly important. Investigating the neuroprotective properties of ZO on neuronal populations in this region may contribute to alternative strategies for mitigating diabetes-induced cerebellar oxidative damage and cellular alterations. This study aimed to evaluate the neuroprotective effects of zingerone on diabetes-induced damage to the central nervous system using a comprehensive biomarker panel. To determine the dose-dependent effects of zingerone on these processes, parameters such as oxidative stress (MDA, SOD, GSH, GR), nitrosative stress (nNOS, iNOS), DNA damage (8-OHdG, H2A.X), apoptosis (Caspase-3), and glial activation (GFAP) were evaluated. The application of NeuN expression, a marker of neuronal integrity, facilitated the comprehensive assessment of structural neuronal degeneration.

## Material and methods

### Ethics approval

Approval from the Atatürk University Animal Experiments Local Ethics Committee was secured for the project (Decision No: 2022/10).

All the authors indicate that all animal experiments comply with the ARRIVE guidelines and are carried out following the U.K. Animals (Scientific Procedures) Act, 1986 and associated guidelines, EU Directive 2010/63/EU for animal experiments, or the National Institutes of Health guide for the care and use of Laboratory animals (NIH Publications No. 8023, revised 1978).

### Chemicals

Streptozotocin (STZ, BioVision Cat No:1930–1000), metformin (MET) (Glifor, 1000 mg, Bilim Medicine), and zingerone (Sigma-Aldrich® Cat No: 88787-50MG) were used.

### Experimental design and animal procedures

The experimental subjects utilized in the study were procured from the Atatürk University Medical Experiment Application and Research Center. Throughout the study, rats were maintained at an ambient temperature of approximately 25 °C in a ventilated setting with a 12-h light–dark cycle and provided with ad libitum access to food. The study utilized 60 adult female Sprague–Dawley rats, aged 12 to 16 weeks and weighing between 220 and 250 g. Rats were randomly allocated into six groups, each including ten animals, and the experimental groups were established as follows: Control group: rats in this group were injected with a single dose of 50 mg/kg saline intraperitoneally (*i.p.*)*.* DM group: The rats in this group were injected with a single dose of STZ (50 mg/kg, *i.p.*). DM + MET group: the rats were given a single dose of STZ (50 mg/kg*, i.p.*), and MET (100 mg/kg, *i.g.*) was administered for 28 days. DM + ZO25 group received a single dose of STZ (50 mg/kg, *i.p.*), and ZO (25 mg/kg, *i.g*.) was administered for 28 days. DM + ZO50 group: The rats were injected single dose of STZ (50 mg/kg, *i.p.*), and ZO (50 mg/kg, *i.g*.) was administered for 28 days. ZO50 group: ZO (50 mg/kg, *i.g.*) was administered for 28 days. The number of animals to be used in the experiments (sample size) was determined by power analysis, where the largest estimated mean body weight difference between groups was 22 g, standard deviation was 10 g, type 1 error (α) was 0.05 and type 2 error (β) (Power = 0.80) and the number of groups was 5; at least 10 animals were required in each group (Minitab for Windows, 16.2.0).

A single dose of 50 mg/kg of intraperitoneal streptozotocin was administered to the groups intended to develop diabetes (Oktay et al. 2024). The blood glucose levels of the rats were evaluated after a 12-h fasting period, 7 days following the application. Rats with blood glucose levels of 140 mg/dL or more were categorized as diabetic.

### Ending the experiment and gathering the tissue samples

The rats received anesthesia via intraperitoneal injection of 8 mg/kg xylazine and 80 mg/kg ketamine on the 28th day post-STZ treatment, and they were euthanized using the cervical dislocation procedure. Histopathological, immunohistochemical (IHC), immunofluorescent (IF), and biochemical (ELISA) examinations were conducted on the extracted brain and cerebellar tissues.

### Histopathological examinations

Brain and cerebellum tissue samples were fixed in 10% buffered formaldehyde for 48 h. After routine tissue follow-up, 5-µm sections were taken from the tissues, and hematoxylin–eosin staining was performed. The histopathological changes were then evaluated under the microscope. Observed histopathological examination was assessed as no (−), very mild (+), mild (+ +), moderate (+ + +), and severe (+ + + +).

### Immunohistochemical examinations

All sections taken on adhesive (poly-L-Lysin) slides for immunoperoxidase examination were deparaffinized and dehydrated by passing through alcohol and xylol series. After the routine procedure, primary antibodies (GFAP Cat No: ab68428, dilution ratio: 1/100, UK, Caspase-3 Cat No: sc-56053, dilution ratio: 1/100, US) were dripped onto the tissues and incubated at 37 °C for 1 h. Then, 3–3′-diaminobenzidine (DAB) chromogen was dropped into the sections as chromogen, and the sections were examined under a light microscope (Zeiss AXIO GERMANY).

### Double immunofluorescence examinations

Tissue sections on poly-L-lysine slides were deparaffinized and dried for immunoperoxidase examination. Endogenous peroxidase was subsequently inactivated in 3% hydrogen peroxide for 10 min. The tissues were then maintained in a 1% antigen retrieval solution (citrate buffer, pH 6.1, 100X) and allowed to cool to ambient temperature. Sections were incubated with a protein block for 5 min to prevent nonspecific background staining. Primary antibodies (nNOS, Cat No: ab16650, 1/100 dilution, UK; NeuN, Cat No: ab104225, 1/100 dilution, UK) were applied to the tissues and incubated according to the manufacturer’s guidelines. Immunofluorescent secondary antibodies (FITC Cat No: ab6785, dilution ratio: 1/500, UK) were utilized as secondary markers. The slides were incubated in the dark for 45 min, after which the second primary antibodies (8-OHdG Cat No: s-66036, dilution ratio: 1/100, US; H2A.X Cat No: I0856-1, Dilution Ratio: 1/100, UK) were applied and incubated according to the manufacturer’s instructions. Subsequently, immunofluorescent secondary antibodies (Texas Red, Cat No. ab6719, dilution ratio 1:500, UK) were used as secondary markers, and the slides were incubated in darkness for 45 min. DAPI (Catalogue no. D1306, dilution ratio: 1/200, UK) is applied to the sections, along with mounting medium, and the tissues are then covered with a coverslip. The dyed tissues were analyzed using a fluorescence microscope (Zeiss AXIO,GERMANY).

### Oxidative stress analyses using enzyme-linked immunosorbent assay (ELISA)

#### Preparation of brain tissue homogenates

Samples of 100 mg were obtained from both brain and cerebellar tissues, placed in 2-ml microtubes, and later augmented with 1.5 ml of phosphate-buffered saline (PBS). Microtubes were put into the Magna Lyser (Roche) homogenizer, and homogenization was performed at 7000 rpm for 80 s. The obtained homogenates were subjected to centrifugation at 15,000 rpm for 10 min at 4 °C. The supernatants were then transferred to sterile containers for biochemical analysis.

### Biochemical analysis

The levels of MDA (Catalogue nr.: 201–11–0157), GSH (Catalogue nr.: 201–11–5134), and iNOS (Catalogue nr.: 201–11–0741), as well as the activities of SOD (Catalogue nr.: 201–11–0169) and GR (Catalogue nr.: 201–11–0552) in brain and cerebellar supernatants, were assessed using commercial ELISA kits according to the manufacturer’s instructions (Sunred Biological Technology, Shanghai, China).

### Statistical analysis

The Kolmogorov–Smirnov test was employed to assess the data distribution, revealing a lack of normality in the results. The nonparametric Kruskal–Wallis test was employed to assess the differences among groups of semiquantitative data derived from histopathological examination, while the Mann–Whitney *U* test was utilized for paired group comparisons. Statistical analyses were conducted using the SPSS 13.0 software package.

To assess the strength of positive staining in photographs acquired using immunohistochemistry and immunofluorescence techniques, five random regions were picked from each image and analyzed using the ZEISS Zen Imaging Software. Data were statistically represented as mean and standard deviation (mean ± SD) for area percentage. A one-way ANOVA, followed by a Tukey test, was conducted to compare positive immunoreactive cells and immunopositive-stained regions with healthy controls. A *p*-value of less than 0.05 was deemed significant, and data are presented as mean ± standard deviation (SD).

The numerical results derived from the biochemical investigations in the study were evaluated using the GraphPad Prism 8.0.1 software. One-way analysis of variance (one-way ANOVA) and the Tukey test are utilized to compare the mean values of multiple independent groups. A *p* value of less than 0.05 was considered statistically significant.

## Results

### Blood glucose levels and live weights

Blood glucose levels and live weights of the animals were measured both at the beginning and at the end of the experiment.

After the induction of diabetes with Stz, the highest blood glucose levels were recorded in the DM, DM+ZO25, and DM+ZO50 groups, and these values were statistically significant compared to the Control and ZO50 groups (*p* < 0.05). The Control and ZO50 groups exhibited no significant change in blood glucose levels (*p* > 0.05). At the conclusion of the study, the DM group exhibited the highest blood glucose levels (*p* < 0.05), whereas the DM+ZO50 group demonstrated values nearest to the control group (Table 1). Although live weight measures were comparable between the groups at the experiment’s onset (*p* > 0.05), the DM group showed a significant decrease in body weight. No statistically significant difference was seen between the Control and ZO50 groups. The DM+ZO50 group exhibited a shift more akin to the control groups than the DM group (Table 2).

**Table 1:** Blood glucose level measurements at the beginning and at the end of the experiment

Groups	Blood glucose level measurements at the beginning of the experiment (mg/dL)	Blood glucose level measurements at the end of the experiment(mg/dL)
Control	96.5 ± 8.11^b^	97.3 ± 7.54^d^
DM	293.5 ± 11.55^a^	301.4 ± 8.16^a^
DM + ZO25	296.9 ± 12.17^a^	197.2 ± 5.85^b^
DM + ZO50	294.2 ± 11.25^a^	132.1 ± 5.85^c^
ZO50	96.9 ± 5.85^b^	98.1 ± 4.28^d^

Values represent mean ± SE (*n* = 10). Across the column, values with superscript different from are significantly different (*p* < 0.05)

**Table 2:** Live weight measurements at the beginning and at the end of the experiment

Groups	Live weight measurements at the beginning of the experiment (gr)	Live weight measurements at the end of the experiment (gr)
Control	235.2 ± 6.52^a^	249.8 ± 4.14^a^
DM	236.5 ± 5.13^a^	189.2 ± 5.13^d^
DM + ZO25	234.9 ± 4.98^a^	211.5 ± 6.53^c^
DM + ZO50	239.2 ± 6.74^a^	224.1 ± 5.17^b^
ZO50	238.9 ± 5.57^a^	244.6 ± 3.94^a^

Values represent mean ± SE (*n* = 10). Across the column, values with superscript different from are significantly different (*p* < 0.05)

### Histopathological results

**Fig. 1:**
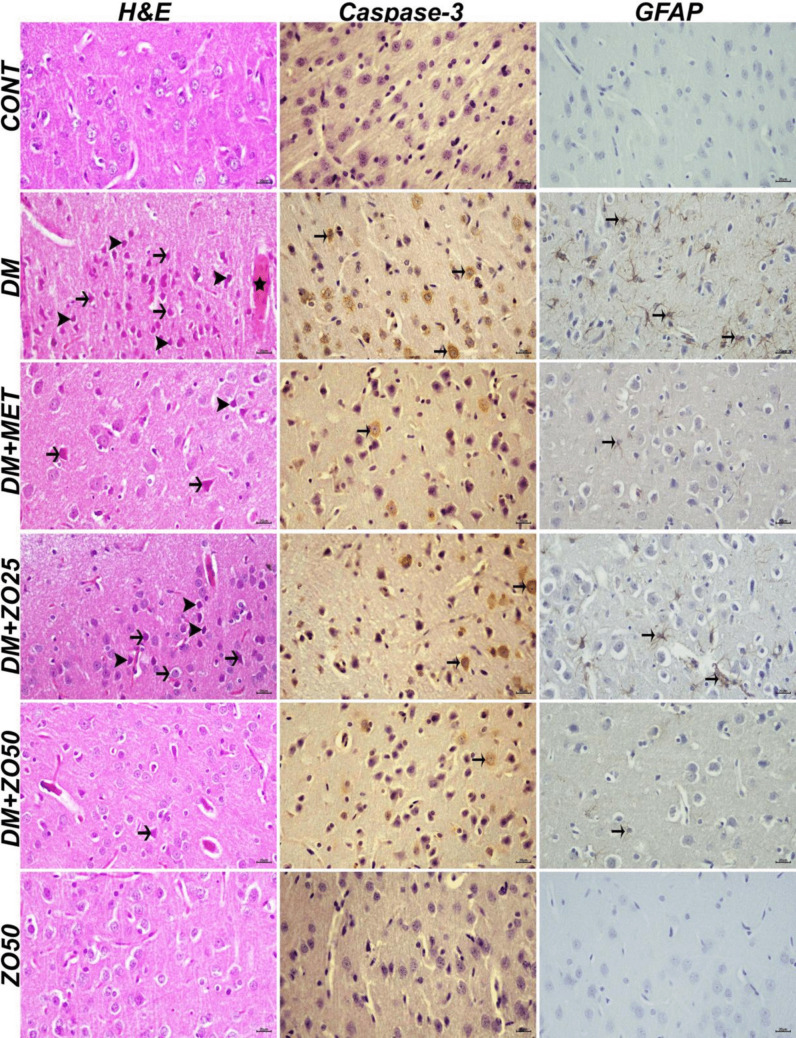
Brain tissue degeneration (arrows) and necrosis (arrowheads) in neurons, hyperemia in vessels (star), H&E, Caspase-3 in neurons, and GFAP expressions in astrocytes (arrow). IHC-P: bar, 20 µm

**Fig. 2:**
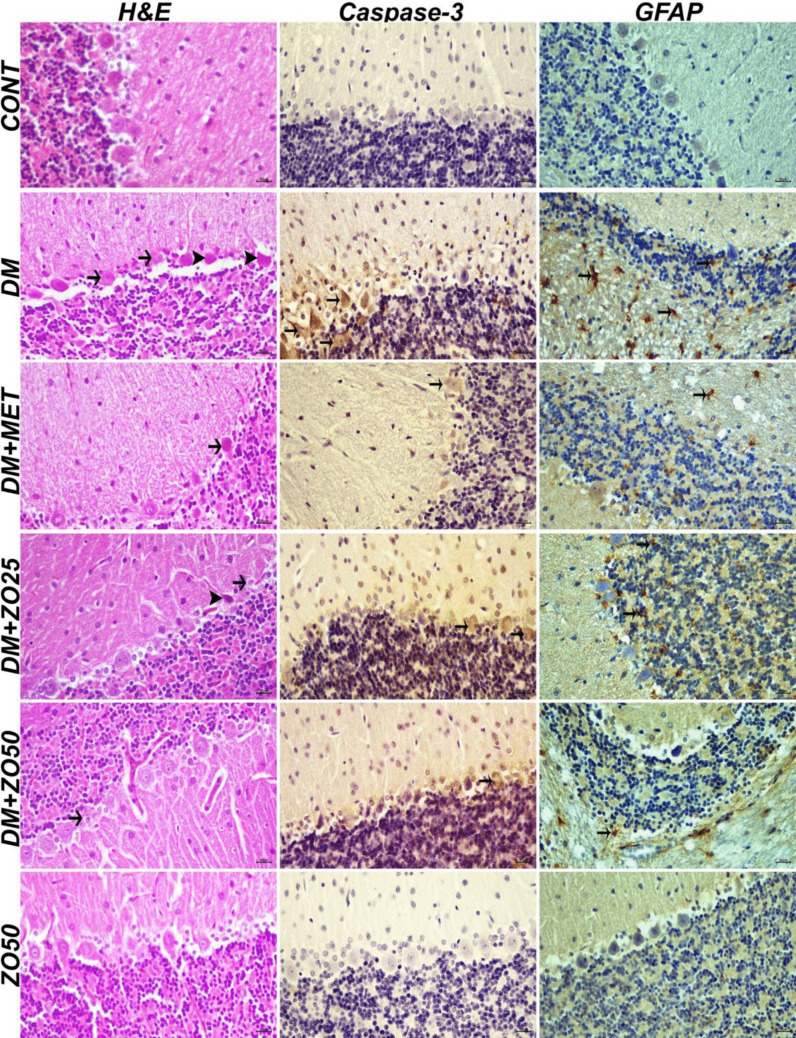
Cerebellum tissue degeneration (arrows) and necrosis (arrowheads) in Purkinje cells, H&E, Caspase-3 in Purkinje cells, and GFAP expressions in astrocytes (arrow), IHC-P: bar, 20 µm

The histopathological examination revealed that the brain and cerebellar tissues of the rats in the control group exhibited a normal histological appearance. Significant degeneration and necrosis were observed in the neurons of the rats in the DM group, accompanied by pronounced hyperemia in the parenchyma and meningeal arteries. In the slides of the brain tissue of the DM + MET group, minor degeneration and slight necrosis in neurons and minor hyperemia in arteries were noted. In contrast, mild degeneration and necrosis were observed in cerebellar tissues and Purkinje cells. A statistically significant difference was observed between the DM + MET and DM groups (*p* < 0.05). In the DM + ZO25 group’s brain tissues, moderate degeneration and necrosis, accompanied by moderate hyperemia in the arteries, were observed. In cerebellar tissues of the DM + ZO25 group, moderate degradation in the overall tissue and necrosis in Purkinje cells were observed. The brain sections of the DM + ZO50 group exhibited mild neuronal degeneration and mild vascular hyperemia. Mild deterioration was observed in the cerebellar tissues and Purkinje cells. A significant difference was observed compared to the DM group (*p* < 0.05). The meninges and parenchyma tissues of the ZO50 group had normal histological features. The results of the histopathological examinations are summarized in Table 3, and representative histological findings are shown in Figs. 1 and 2.

**Table 3:** Scoring of histopathological findings in the brain and cerebellum tissues of rats in the experimental groups

Experimental groups	Degeneration in neurons	Necrosis in neurons	Hyperemia in the veins	Damage to Purkinje cells
Control	−	−	−	−
DM	+ + + +	+ + + +	+ + + +	+ + + +
DM + MET	+ +	+	+ + +	+ +
DM + ZO25	+ + +	+ + +	+ + +	+ + +
DM + ZO50	+ +	−	+ + +	+
ZO50	−	−	−	−

#### Immunohistochemical results

The immunohistochemical analyses of brain and cerebellar tissues from the control and ZO50 groups demonstrated negative immunoreactivity for Caspase-3 and GFAP antibodies. The brain tissues of the DM group exhibited pronounced cytoplasmic Caspase-3 expression in neurons and significant GFAP expression in astrocytes. Marked cytoplasmic Caspase-3 expression was detected in cerebellar tissues, especially within Purkinje cells, while pronounced GFAP expression was predominantly identified in astrocytes in the substantia grisea of the DM group. Mild amounts of Caspase-3 immunoreactivity are observed in neurons of the DM + MET group brain tissues and Purkinje cells of cerebellar tissues. GFAP expression was observed to be triggered at a moderate level in astrocytes in the brain and cerebellar tissues. Moderate activation of cytoplasmic Caspase-3 was observed in neurons of brain tissues and in Purkinje cells of the cerebellum in the DM + ZO25 group.

It was found to express moderate GFAP in the brain and cerebellum tissues and astrocytes. Mild cytoplasmic Caspase-3 expression was detected in neurons in the brain tissues of the DM + ZO50 group and in Purkinje cells in the cerebellum tissues. In the brain and cerebellum tissues, mild GFAP expression was detected in astrocytes (Figs. 1 and 2). Immunohistochemical analysis results and statistical findings are presented in Table 4.

**Table 4:** Statistical analysis results of immunohistochemistry and immunofluorescence results in brain tissues

Experimental groups	Caspase-3 W/sr	GFAP W/sr	nNOS W/sr	8-OHdG W/sr	NeuN W/sr	H2A.X W/sr
Control	21.56 ± 0.68^a^	17.14 ± 1.48^a^	24.19 ± 0.45^a^	26.16 ± 0.26^a^	25.18 ± 0.16^a^	22.19 ± 0.14^a^
DM	79.89 ± 1.64^b^	64.45 ± 2.49^b^	84.76 ± 1.19^b^	88.19 ± 1.85^b^	86.75 ± 1.19^b^	81.16 ± 2.15^b^
DM + MET	32.15 ± 1.4^c^	28.16 ± 1.15^c^	34.15 ± 1.85^c^	37.19 ± 1.16^c^	39.74 ± 1.16^c^	36.12 ± 1.49^c^
DM + ZO25	56.14 ± 7.15^d^	51.18 ± 1.37^d^	68.92 ± 1.76^d^	69.26 ± 1.72^d^	67.16 ± 1.72^d^	68.74 ± 1.76^d^
DM + ZO50	31.10 ± 1.94^c^	30.26 ± 1.84^c^	36.76 ± 1.15^c^	39.41 ± 0.84^c^	40.18 ± 1.15^c^	38.76 ± 1.12^c^
ZO50	20.41 ± 0.68^a^	15.12 ± 0.16^a^	26.74 ± 0.86^a^	27.18 ± 0.15^a^	27.15 ± 0.15^a^	24.71 ± 0.49^a^

Values represent mean ± SE (*n* = 10). Across the column, values with superscript different from are significantly different (*p* < 0.05). W/sr (unit of luminous intensity)

### Immunofluorescence results

**Fig. 3:**
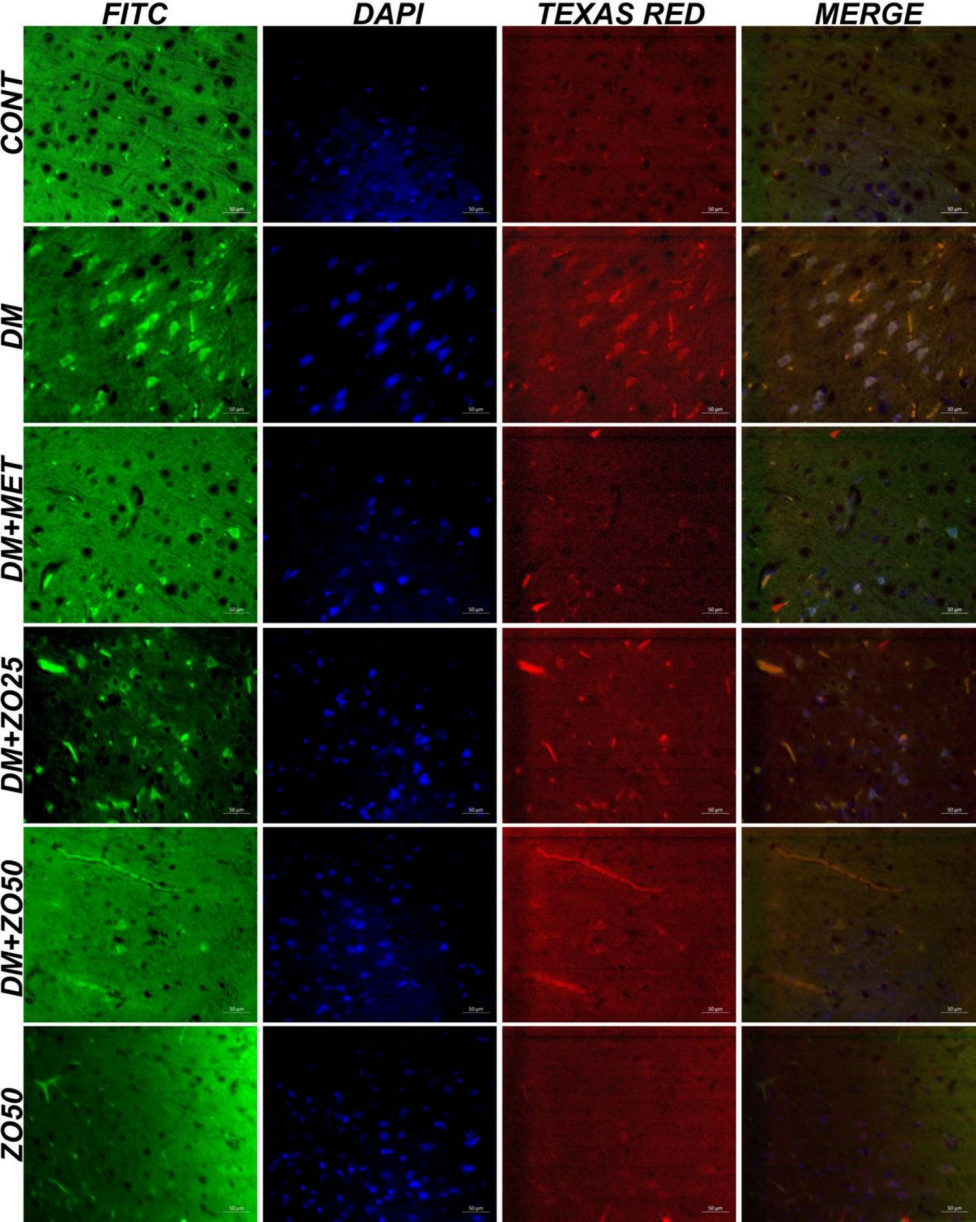
Brain tissue; nNOS (FITC) and 8-OHdG (Texas Red) expressions in neurons. IF: bar, 50 µm

**Fig. 4:**
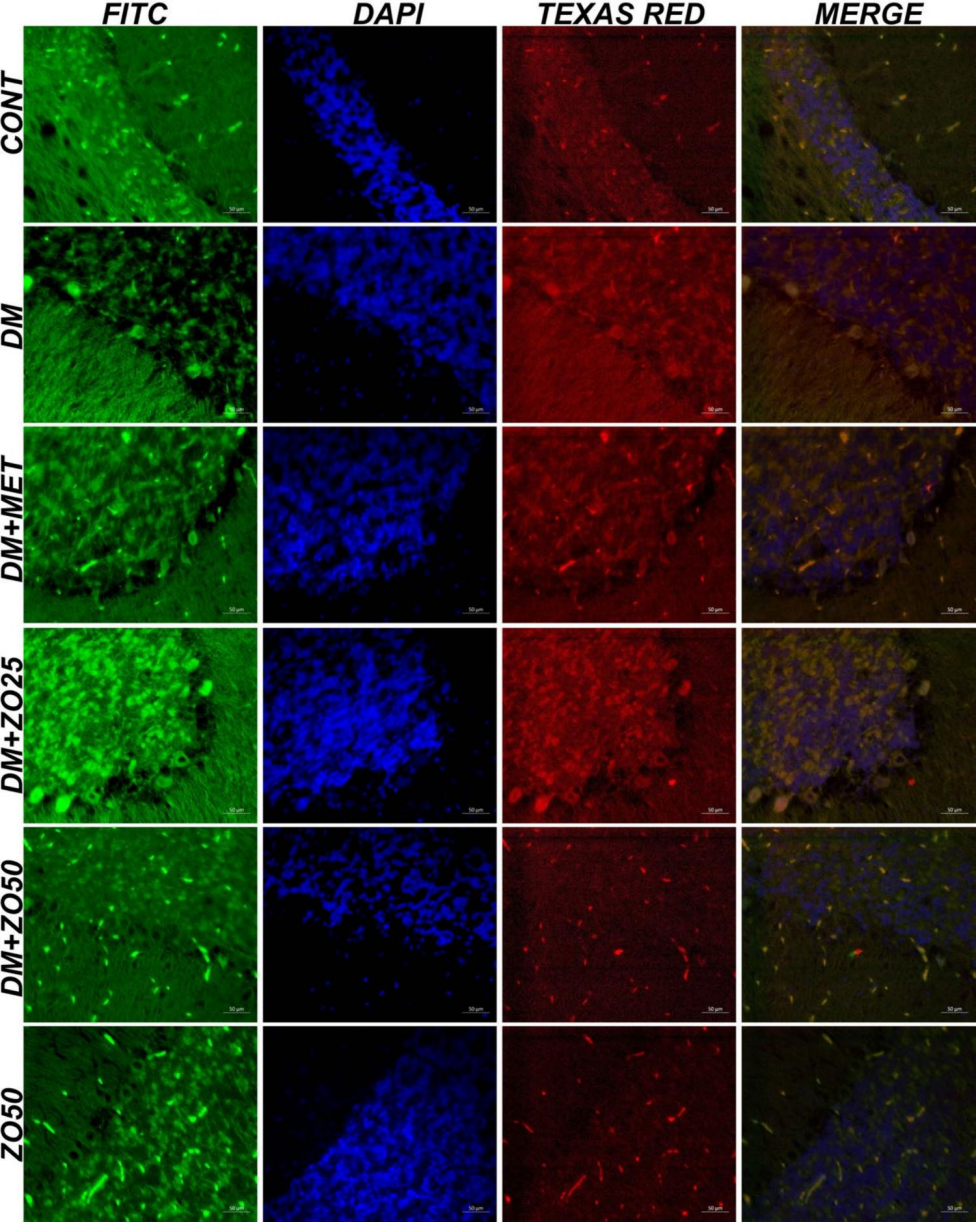
Cerebellum tissue; nNOS (FITC) and 8-OHdG (Texas Red) expressions in Purkinje cells. IF: bars, 50 µm

**Fig. 5:**
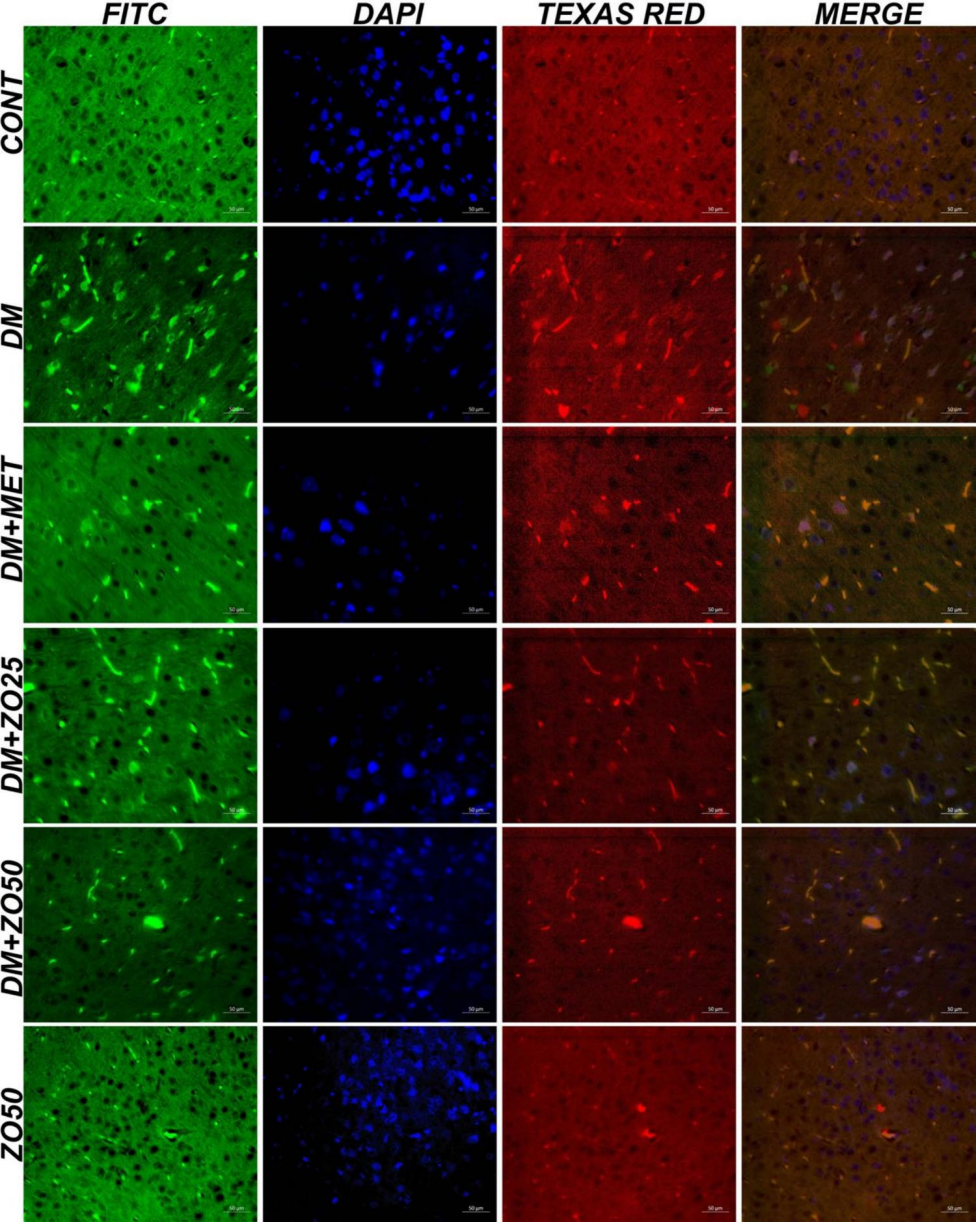
Brain tissue; NeuN (FITC) and H2A.X (Texas Red) expressions in neurons. IF: bars, 50 µm

**Fig. 6:**
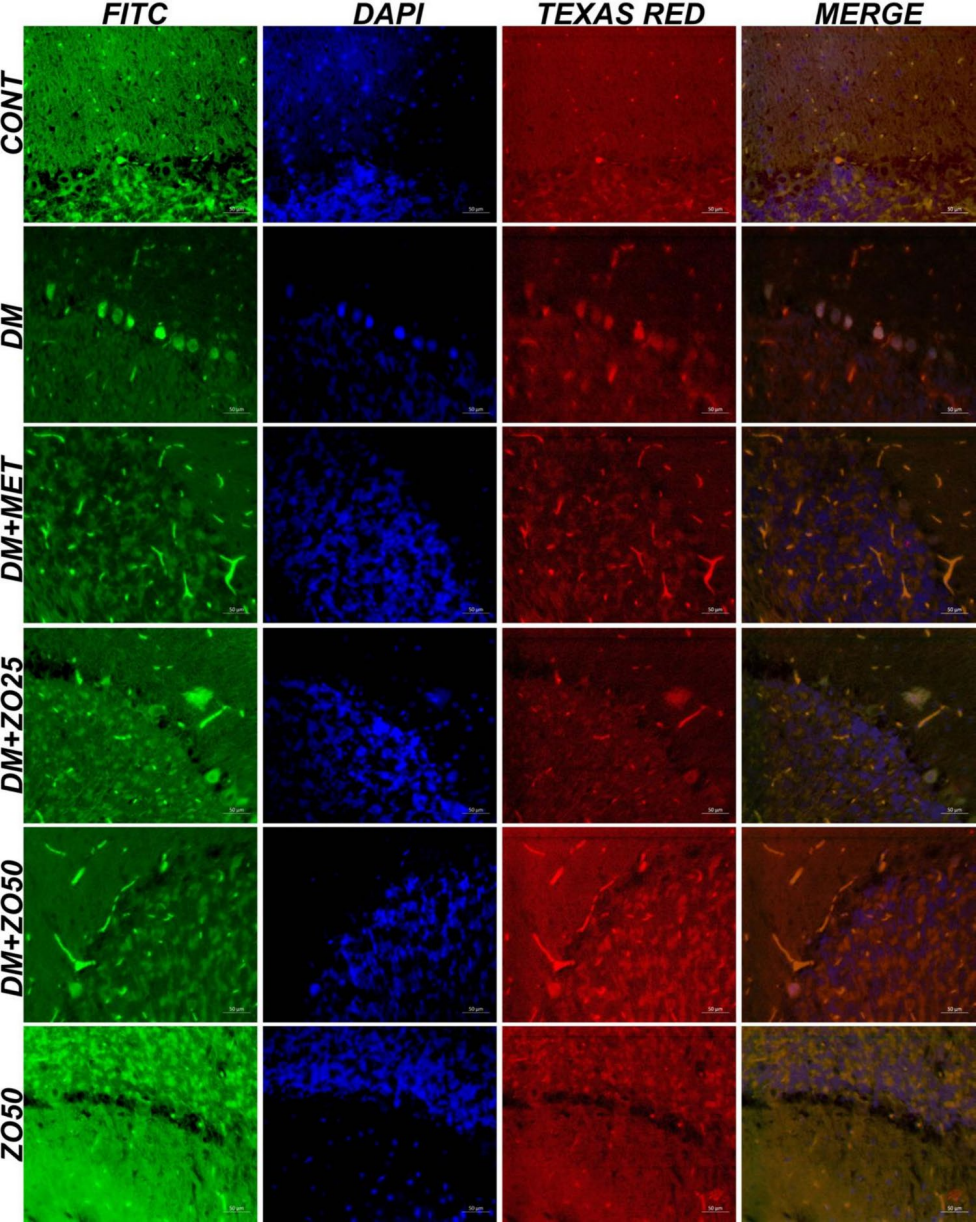
Cerebellum tissue; NeuN (FITC) and H2A.X (Texas Red) expressions in Purkinje cells. IF: bars, 50 µm

The brain and cerebellum tissues of rats in the control and ZO50 groups were examined by the immunofluorescence method, and nNOS, 8-OHdG, NeuN, and H2A.X expressions were evaluated as negative. Severe cytoplasmic nNOS, 8-OHdG, NeuN, and H2A.X expressions were detected in neurons in the brain tissues of the DM group and in Purkinje cells in the cerebellum tissues. Mild nNOS, 8-OHdG, NeuN, and H2A.X expressions were detected in neurons in the brain tissues of the DM + MET group and in Purkinje cells in the cerebellum tissues. nNOS, 8-OHdG, NeuN, and H2A.X expressions were detected in the brain tissues of the rats in the DM + ZO50 group, in the neurons, and in the cerebellum tissues in the Purkinje cells at moderate levels. Analysis results and statistical findings of immunofluorescent staining are presented in Figs. 3, 4, 5, and 6 and Tables 4 and 5.

**Table 5:** Statistical analysis results of immunohistochemistry and immunofluorescence findings in cerebellum tissues

Experimental groups	Caspase-3 W/sr	GFAP W/sr	nNOS W/sr	8-OHdG W/sr	NeuN W/sr	H2A.X W/sr
Control	18.59 ± 0.26^a^	15.45 ± 0.02^a^	21.64 ± 0.1^a^	23.59 ± 0.51^a^	24.97 ± 0.23^a^	25.12 ± 0.18^a^
DM	55.71 ± 2.20^b^	51.19 ± 1.51^b^	85.48 ± 3.12^b^	84.76 ± 3.71^b^	92.64 ± 4.67^b^	95.58 ± 3.58^b^
DM + MET	28.16 ± 0.63^c^	25.3 ± 0.39^c^	37.59 ± 0.61^c^	35.90 ± 1.59^c^	41.83 ± 1.59^c^	44.06 ± 1.44^c^
DM + ZO25	42.59 ± 1.00^d^	37.66 ± 1.43^d^	69.71 ± 1.18^d^	66.66 ± 1.97^d^	77.58 ± 2.61^d^	78.00 ± 2.33^d^
DM + ZO50	26.00 ± 0.3^c^	24.87 ± 0.48^c^	32.48 ± 0.39^c^	33.10 ± 0.41^c^	43.19 ± 1.57^c^	43.55 ± 1.26^c^
ZO50	18.66 ± 0.18^a^	15.58 ± 0.15^a^	22.50 ± 0.10^a^	22.87 ± 0.38^a^	25.41 ± 0.48^a^	25.00 ± 0.12^a^

Values represent mean ± SE (*n* = 10). Control across the column, values with superscript different from a are significantly different (*p* < 0.05). W/sr (unit of luminous intensity)

### Effects of ZO and DM on oxidative stress parameters in brain tissues

**Fig. 7:**
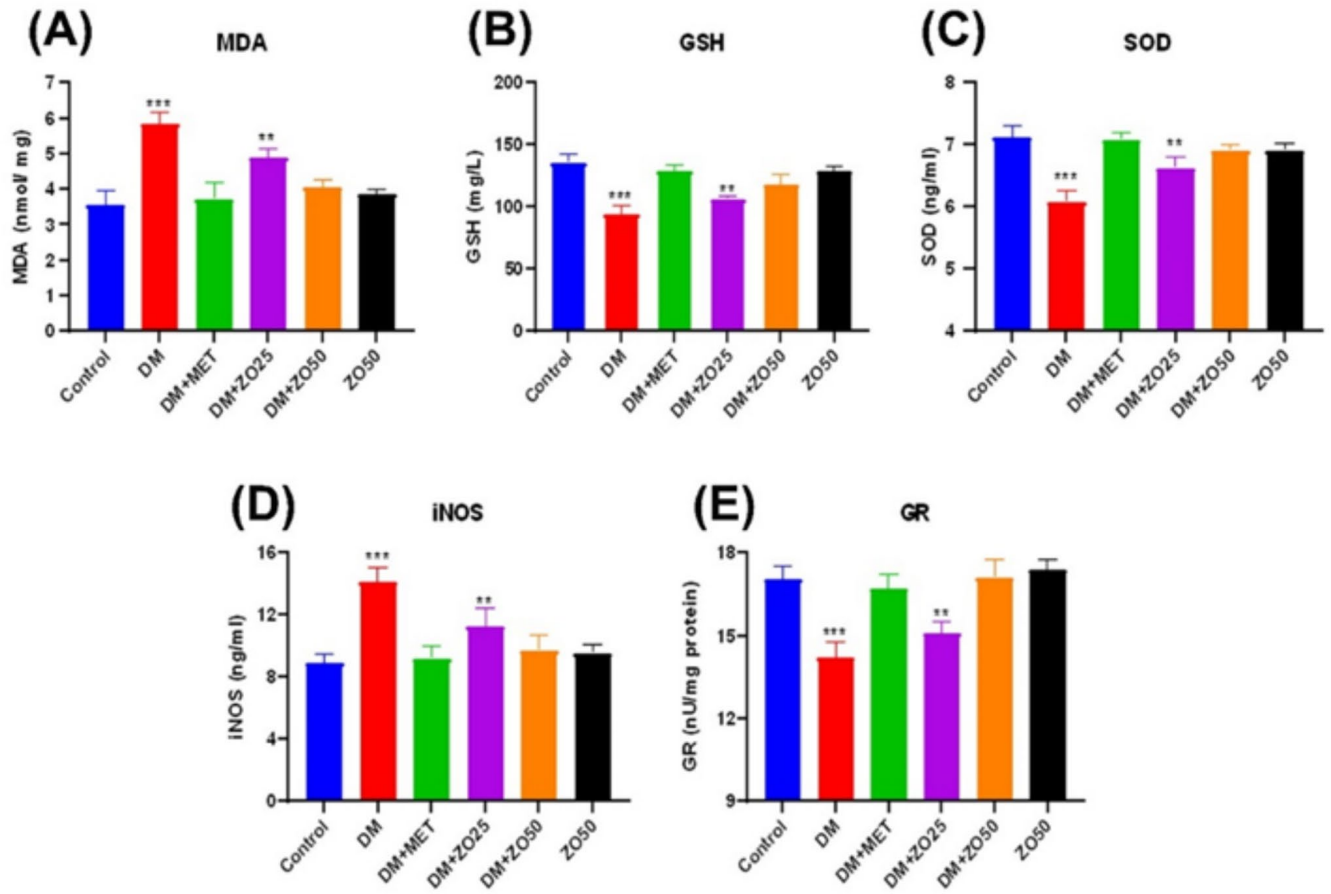
The effect of zingerone (ZO), metformin (MET), and diabetes mellitus (DM) on malondialdehyde (MDA) (**A**), glutathione (GSH) (**B**), superoxide dismutase (SOD) (**C**), inducible nitric oxide synthetase (iNOS) (**D**), and glutathione reductase (GR) (**E**) levels in the brain of the rat. Each bar with vertical stripes represents the mean value ± SEM compared to the control (***p < 0.01, **p < 0.05, *n* = 10)

Lipid peroxidation in the brain tissues of rats was found to be significantly increased in the DM and DM + ZO25 groups compared to the other groups (*p* < 0.05). ZO (50 mg/kg) was associated with a significant reduction in MDA levels (*p* < 0.05) (Fig. 7A). The levels of GSH (Fig. 7B) and the activity of SOD (Fig. 7C) and GR (Fig. 7E) were considerably diminished in the DM and DM + ZO25 groups in comparison to the control and other groups (*p* < 0.05). A 50 mg/kg dosage of ZO substantially inhibited the reduction in the levels and activity of these enzymes (*p* < 0.05). The iNOS levels in the brain tissues of the DM and DM + ZO25 groups were significantly elevated (*p* < 0.05), but the iNOS levels in the DM + MET, DM + ZO50, and ZO50 groups did not vary from the control (*p* > 0.05).

## Discussion

Chronic hyperglycemia is widely recognized to have detrimental consequences, especially on the central nervous system, by inducing oxidative and nitrosative stress and apoptosis. It is well known that STZ, commonly used to create a type 1 diabetes model, increases ROS levels and disrupts the antioxidant system in favor of oxidants. This study aimed to evaluate the neuroprotective effects of ZO in the brain and cerebellar tissues of type 1 diabetic rat model induced by STZ, using multiple biochemical, histological, immunofluorescence, and immunohistochemical parameters.

Oxidative stress-induced lipid peroxidation leads to elevated MDA levels, a detrimental consequence, while GSH is crucial in mitigating the negative effects of reactive oxidant species. Elevated MDA levels indicate that membrane lipids are damaged by reactive oxygen species, negatively impacting cell membrane integrity. This can lead to impaired synaptic transmission and cellular integrity. Decreased SOD, GR, and GSH levels indicate inadequate endogenous defense mechanisms and that the oxidative load exceeds the intracellular buffering capacity. The decrease in natural antioxidants makes brain cells vulnerable, and structural abnormalities caused by oxidative stress progress rapidly.

Our investigation revealed a considerable increase in MDA levels and a notable decrease in GSH, SOD, and GR levels in the brain and cerebellum tissues of rats subjected to STZ. A study investigated oxidative stress indicators in the brain and hippocampus of rats with STZ-induced diabetes, revealing elevated MDA levels and diminished SOD and CAT levels in the affected tissues (Marefati et al. 2022). Another study indicated increased oxidative stress in brain tissue in STZ-induced type 1 diabetes (Bathina and Das 2021), and in another study, researchers reported that MDA levels, which are oxidative stress markers, increased and GSH levels decreased in the brain tissues of STZ-induced type 1 diabetic rats (Aydın et al. 2021). Our study showed that in both brain and cerebellar tissues of the DM group, MDA levels were increased, and antioxidant activity (SOD, GSH, GR) was decreased compared to the control group, consistent with previous reports..

NO generated by iNOS and nNOS contributes to the activation of cyclooxygenase, particularly in brain inflammation induced by nNOS. Increased concentrations of nNOS and iNOS, prevalent throughout the nervous system, are responsible for modulating synaptic transmission and neuroplasticity, nitrosative stress, and inflammation in the brain (Dincel and Yildirim 2016; Iova et al. 2023). The elevation of iNOS indicates the production of inflammatory nitric oxide, primarily created by macrophages and glial cells (Chen 2024). Elevated NO synthesis by iNOS leads to persistent inflammation and triggers the generation of strong oxidants (Andrabi et al. 2023). A toxicology research found elevated levels of nNOS and iNOS in brain tissue due to mercury chloride-induced neurotoxicity (Akaras et al. 2025). Similarly, research indicates that ischemia-induced hypoxia elevates nNOS and iNOS levels in cerebral tissue (Anwar et al. 2025). It is also shown that diabetes caused higher expressions of nNOS and iNOS in the cerebellar tissue (Elhessy et al. 2020). Increased nNOS levels indicate that excessive neuronal NO generation adversely affects mitochondrial function and modifies energy expenditure. Our findings revealed that nNOS expression and iNOS levels were markedly elevated in the brain and cerebellar tissues of the DM group in comparison to the control group. These findings indicate that DM is associated with increased nitrosative stress and inflammation. The concurrent increase in oxidative stress markers in the tissues of the same group indicates that hyperglycemia linked to diabetes mellitus impairs several cellular activities.

Evidence suggests that H2A.X, a protein indicative of DNA breaks, is elevated in multiple tissues in individuals with diabetes (Hiramatsu et al. 2017; Xiang et al. 2024; Yang et al. 2024). Our work demonstrated that the assessment of H2A.X immunofluorescence labeling in brain and cerebellum tissues from the DM group exhibited a pronounced immune response to both antibodies. The observations indicate DNA-related damage in brain and cerebellum tissue due to type 1 diabetes, aligning with existing research. On the other hand, DNA oxidation is essential for the onset of persistent neuronal damage and neurodegenerative processes. Oxidative damage to mitochondrial DNA further undermines brain energy expenditure. Elevated levels of 8-OHdG, a robust marker of DNA damage, have been correlated with markedly compromised genomic integrity in prediabetic animal model (Al-Aubaidy and Jelinek 2010). These findings suggest that ZO may reduce 8-OHdG levels, potentially contributing to protection against radical damage and aiding in the maintenance of DNA integrity. Literature increasingly indicates that zingerone may enhance DNA repair pathways (Çiğ et al. 2026). NeuN functions as a crucial marker for assessing neuronal injury and the preservation of neuronal integrity (Yuan et al. 2024). Elevated NeuN levels have been extensively documented in diabetes research (Lo et al. 2016; Oliveira et al. 2016). Our work identified NeuN expression using immunofluorescence labeling, revealing a substantial elevation in the DM group. The reduction in NeuN expression in the diabetic animals treated with ZO (DM + ZO group—50 mg/kg) indicated that neuronal integrity and cellular architecture were maintained.

In this study, metformin (dimethyl biguanide), which is frequently used to lower blood sugar levels in individuals with non-insulin-dependent diabetes (Herman et al. 2022), was used as a conventional oral antidiabetic agent. Research indicates its efficacy in regulating blood glucose levels with a minimal danger of hypoglycemia; still, prevalent adverse effects encompass nausea, diarrhea, abdominal discomfort, and vitamin B12 deficiency (Lv and Guo 2020; Al Quran et al. 2023). From these views, despite its beneficial effects and years of efficacy in diabetes therapy, there is a pursuit for alternative therapeutic drugs in patient populations with inadequate tolerance to metformin. The 100 mg/kg metformin dosage is the chosen regimen as it has set a therapeutic benchmark for diabetes produced in rats across numerous investigations (El-Ashmawy et al. 2018; Hacioglu et al. 2021; Indla et al. 2023). Oxidative and metabolic stress induced by hyperglycemia is identified as the principal cause of tissue damage. The harm inflicted by free radicals cannot be alleviated without augmenting the amounts of cytoprotective enzymes and supplementary antioxidants. From this viewpoint, alongside metformin, recognized as the conventional treatment for diabetes, it may be beneficial to enhance metabolism with specific molecules abundant in antioxidants and cytoprotective enzymes.

ZO is a phenolic molecule recognized for its antioxidant and cytoprotective properties, and its efficacy was assessed in our study using two distinct dosages (25 and 50 mg/kg). Numerous experimental investigations in the literature indicate that ZO exhibited ameliorative effects on oxidative stress and inflammatory markers when supplied at dosages of 25 and 50 mg/kg (Kandemir et al. 2025). Furthermore, papers indicate that oxidative stress caused by diverse brain tissue damage is mitigated with ZO administration (Vaibhav et al. 2013; Banji et al. 2014; Upadhyaya et al. 2022; Chopra et al. 2023; Oviosun et al. 2025). Moreover, research indicates that ZO may function as an antidiabetic agent (Rehman et al. 2019; Singh et al. 2020; Anwer et al. 2021; Mokhtare and Saglam 2025; Utlu et al. 2026). The administered doses are suitable for assessing the dose–response relationship, and research comparing the 25 and 50 mg/kg groups has demonstrated a dose-dependent increase in therapeutic effect (Şimşek et al. 2023). Moreover, these doses remain inside the safety threshold, avoiding a significant risk of toxicity; nonetheless, elevated doses (e.g., 100 mg/kg) have been documented to correlate with minor toxic effects in certain investigations (Şimşek et al. 2023). In our study, the results indicated that ZO50 administration in diabetic rats yielded outcomes for diabetic and oxidative stress parameters comparable to those achieved with metformin.

Upon evaluating the results of our study, we observed that the ZO 25 mg/kg dosage (DM + ZO 25 mg/kg group) administered to diabetic rats was therapeutically inadequate concerning both histological and biochemical parameters, including oxidative damage, inflammation, cell density indicative of apoptosis, and cells exhibiting DNA damage in the brain and cerebellum tissues of STZ-induced diabetes. The outcomes achieved with the DM + ZO 50 mg/kg dosage were comparable to those of the DM + MET (therapeutic control) groups, suggesting potential relevance of ZO in diabetes-related neurotoxicity. The diminution of MDA levels by ZO is essential for preserving membrane integrity. The lipophilic characteristics of ZO may elucidate its capacity to safeguard membrane lipids from oxidative harm (Mani et al. 2016; Geng et al. 2021; Motamedi et al. 2024). When assessed alongside other oxidative damage markers, the observation that the ZO dosage administered in the DM + ZO 50 mg/kg group brought these parameters near the levels of the therapeutic control group may suggest that these phenolic compounds not only neutralize radicals but also enhance the production of antioxidant enzymes.

Our study, based on results obtained from tissue analysis of the DM + ZO 50 mg/kg group, shows that it leads to a decrease in nNOS and iNOS enzyme levels in both the brain and cerebellum tissues. This indicates that ZO may exert broad antioxidant effects, potentially modulating both oxidative and nitrosative pathways. Prior research has demonstrated that phenolic compounds can interact with nitric oxide to diminish detrimental nitric oxide molecules (Gungor et al. 2020; Olasehinde and Olaokun 2025). Although our work aligns with the pertinent literature, there is a lack of evidence on the impact of ZO 50 mg/kg on nNOS and iNOS levels in the brain and cerebellum tissues of type 1 diabetic rats. In this context, these findings may contribute to addressing an existing gap in the literature.

Caspase-3 and GFAP are recognized as significant markers and early indicators of damaged tissue responses (Günther et al. 2017). Immunohistochemical evaluations of Caspase-3 and GFAP expression in target tissues have been performed to elucidate the effect of ZO on diabetes-induced brain and cerebellum damage. There is no research on the direct application of ZO in the analysis of Caspase-3 and GFAP expression in diabetic neuropathy. However, El-Akabawy et al. investigated the short- and long-term efficacy of ginger use against diabetic neurodegeneration and examined Caspase-3 and GFAP expressions. According to their findings, the use of ginger reduced Caspase-3 and GFAP expressions (El-Akabawy and El-Kholy 2014). Badawy et al. stated that ZO reduced Caspase-3 expression in neurotoxicity induced by gabapentin (Badawy et al. 2019). In the study, the anti-apoptotic effect of ZO, which decreases Caspase-3 expression, explains its role in preventing and healing neuronal tissue damage caused by diabetes. Previous experimental studies also suggest that modulation of apoptotic pathways, particularly caspase-3 signaling, represents an important component of the protective effects of several pharmacological agents in different experimental injury models. For instance, memantine has been reported to attenuate risperidone-induced dysfunction in rats, an effect accompanied by a reduction in caspase-3 expression together with modulation of ERK1/2–Nrf2 signaling pathways (Mohyeldin et al. 2025). Similarly, buspirone was shown to ameliorate cyclophosphamide-induced damage in an experimental model of premature ovarian insufficiency, where its protective actions were associated with the regulation of oxidative stress-related pathways such as AMPK/Nrf2/HO-1 and α-Klotho/NLRP3 signaling and a concomitant decrease in caspase-3-mediated apoptosis (Khallaf et al. 2025). Although these studies were conducted in different experimental contexts, they collectively support the concept that attenuation of caspase-3 activation may represent a common mechanism underlying the protective actions of various compounds against experimentally induced cellular injury. A study indicated that the injection of fluorocitrate and neurotropin diminished elevated GFAP expression in diabetic rats via suppressing astrocyte activation (Liu et al. 2022). A study examining the neuroprotective effects of semaglutide and metformin in diabetic rats found that metformin treatment resulted in notable decreases in Caspase-3 and GFAP expression (Salem et al. 2025). In our study, we also observed that ZO administration, similar to the effect of MET, reduced the high expressions of GFAP and Caspase-3 in the brain and cerebellar tissues associated with diabetes.

This study revealed extensive neuronal degeneration and necrosis, along with pronounced hyperemia in meningeal and parenchymal tissue, in the DM group. The injection of ZO demonstrated a protective impact on alterations in brain tissue, contingent upon the dosage provided. Moreover, the 50 mg/kg dosage of ZO provided to diabetic rats in our investigation exhibited a protective effect comparable to the therapeutic efficacy of MET. The histopathological damage score was higher in both tissues of STZ-administered rats, and it was determined that ZO (50 mg/kg) administration decreased the damage score in diabetic animals.

These findings collectively indicate that ZO appears to provide protective effects against diabetes-related neurotoxicity against diabetes-related neurotoxicity. Due to its impact on oxidative stress, nitrosative stress, inflammation, and apoptosis, ZO may function as a promising natural adjunct in the future treatment of diabetes-induced neurodegeneration; however, additional research is required to draw therapeutic conclusions.

On the other hand, this study has some limitations. The first of these is that only female rats were used in the study. Rebolledo-Solleiro and Fernández-Guasti highlighted that STZ established a reliable diabetes model in female rats with an active estrous cycle, and that metabolic parameters remained constant across different cycle phases (Rebolledo-Solleiro and Fernández-Guasti 2018). This finding indicates that hormonal fluctuations do not adversely affect the model’s validity and that female rats are suitable for diabetes research. Moreover, the predominance of male rats in studies employing STZ-induced diabetes models underscores a notable deficiency in the literature. This study, conducted solely on female rats, addresses a gender disparity in the literature and offers more extensive data on the biological effects of ZO.

A further disadvantage of the study is the absence of functional or behavioral assessments. This study concentrated on the impact of diabetes on the central nervous system, as opposed to the neuropathy it induces in the peripheral nervous system, and examined these evaluations within a cellular and tissue framework utilizing histopathological, immunohistochemical, immunofluorescent, and biochemical methodologies. In addition, the study was conducted at a single experimental time point (28 days), which restricts the assessment of temporal changes in oxidative stress, inflammation, and apoptosis during disease progression. Furthermore, although multiple oxidative, inflammatory, and apoptotic markers were evaluated, specific intracellular signaling pathways such as Nrf2/Keap1, SIRT1, or NF-κB were not directly investigated; therefore, the precise molecular mechanisms underlying the observed effects remain to be clarified. Another limitation is the lack of pharmacokinetic evaluation of zingerone, as serum or tissue concentrations were not measured, preventing the establishment of a direct relationship between administered dose and systemic exposure. Finally, although the findings provide experimental evidence, their translational relevance to human diabetes requires further investigation.

The findings of this study align with those of other published research, indicating that ZO exerts a therapeutic impact on neuronal tissue damage and inhibits the progression of degenerative alterations in the brain and cerebellum within a diabetic neurotoxicity model.

## Data Availability

The data that support the findings of this study are available from the corresponding author, GCD and SY, upon reasonable request.
